# Workplace Violence Exposure and Burnout Among Fire-Service-Based Paramedics in South Korea: The Indirect Association Through Post-Traumatic Stress Symptoms and the Moderating Roles of Social Support and Resilience

**DOI:** 10.3390/healthcare14213607

**Published:** 2026-10-24

**Authors:** Nayoon Lee, Mihyun Kim

**Affiliations:** 1 College of Nursing, Dong-A University, G11-517, 32-7 Mangyang-ro 111, Seo-gu, Busan 49201, Republic of Korea; 095750@dau.ac.kr; 2 Graduate School, College of Nursing, Dong-A University, G11-517, 32-7 Mangyang-ro 111, Seo-gu, Busan 49201, Republic of Korea

**Keywords:** workplace violence, paramedics, post-traumatic stress symptoms, burnout, social support, resilience

## Abstract

**Highlights:**

**What are the main findings?**

- Workplace violence exposure was indirectly associated with burnout through post-traumatic stress symptoms among Korean fire-service-based paramedics.
- Social support moderated the associations of workplace violence exposure with post-traumatic stress symptoms and burnout.
- The conditional indirect association through post-traumatic stress symptoms decreased as social support increased, with a significant index of moderated mediation.
- Evidence for moderation by resilience was less robust, and the index of moderated mediation for resilience was not statistically significant.

**What are the implications of the main findings?**

- Social support may be an important interpersonal resource in the association between workplace violence exposure and psychological outcomes among fire-service-based paramedics.
- Organizational efforts to reduce workplace violence should be accompanied by strategies to strengthen interpersonal support and resilience-related resources.
- Longitudinal studies are needed to clarify the temporal relationships suggested by these cross-sectional findings.

**Abstract:**

**Background/Objectives:** This study examined the indirect association between workplace violence exposure and burnout through post-traumatic stress symptoms (PTSS) and the moderating roles of social support and resilience among South Korean fire-service-based paramedics. **Methods:** This analytical cross-sectional study included 210 paramedics in Busan, South Korea. Conditional process analyses were conducted using PROCESS Model 8, with social support and resilience examined in separate models. **Results:** Workplace violence exposure was positively associated with PTSS, which was positively associated with burnout. Conditional indirect associations were significant at the mean of each moderator. In the final sample (N = 210), social support moderated the associations of workplace violence exposure with PTSS (*p* = 0.002) and burnout (*p* = 0.015), and its index of moderated mediation was significant (−0.024, 95% bootstrap CI [−0.041, −0.009]). However, the social support interaction predicting PTSS and its index of moderated mediation were statistically significant only after excluding one influential observation (N = 210 vs. N = 211). The social support interaction predicting burnout remained significant in both samples. Resilience did not significantly moderate the PTSS association; its nominally significant interaction predicting burnout did not remain significant after Holm correction, and its index of moderated mediation was not significant. **Conclusions:** Social support may play a moderating role in the associations linking workplace violence exposure, PTSS, and burnout, although some findings were sensitive to the exclusion of one influential observation. These cross-sectional findings require cautious interpretation and further replication.

## 1. Introduction

### 1.1. Background

In South Korea, the national emergency call number is 119, and paramedics working within the fire-service-based emergency medical system are commonly referred to as fire-service-based paramedics. These paramedics are essential personnel in the emergency medical services (EMS) system, providing emergency care from the scene of an incident through transportation to the hospital to protect the lives and safety of patients [1]. However, because they are directly exposed to a wide range of emergency situations while working 24-hour shifts and frequently interact with patients and their caregivers, they are considered a group at high risk of workplace violence [2]. Indeed, violent incidents involving paramedics continue to occur, with repeated reports of violence perpetrated by patients and their caregivers [3,4].

Repeated exposure to such violence may result not only in physical injury but also in psychological trauma responses [5]. Post-traumatic stress symptoms, which include emotional instability, hyperarousal, and avoidance, can adversely affect occupational functioning and have been identified as an important factor associated with burnout [6]. Burnout is a state of emotional, physical, and mental exhaustion resulting from chronic occupational stress. It may reduce job satisfaction and organizational commitment, increase turnover intention, impair work performance, and ultimately compromise the quality of emergency medical services [7,8].

Not all paramedics exposed to similar forms of violence experience the same levels of post-traumatic stress symptoms or burnout, suggesting that these associations may vary according to the psychological and social resources available to individuals [9]. In the present study, social support refers to perceived support from family, friends, and significant others [10] and represents an important psychosocial resource that may buffer the adverse association between stress and psychological well-being [11].

Resilience, meanwhile, refers to an individual’s internal capacity to maintain psychological balance and recover in the face of trauma or stressful circumstances [12,13]. Resilience has been suggested as an important personal resource associated with lower levels of post-traumatic stress symptoms and burnout [14]. Accordingly, social support and resilience warrant consideration as important protective resources that may buffer the associations among workplace violence exposure, post-traumatic stress symptoms, and burnout.

Previous studies have primarily focused on the relationship between workplace violence exposure and burnout among paramedics or on indirect associations involving factors such as job stress [15,16]. Earlier research among Korean 119 rescue workers also documented a positive association between workplace violence exposure and burnout [17]. More recently, a study of Korean paramedics examined the direct and indirect pathways among workplace violence exposure, post-traumatic stress symptoms, resilience, and burnout using path analysis, including the indirect association between workplace violence exposure and burnout through post-traumatic stress symptoms; however, it did not examine resilience as a moderator [18]. Another study examined family, coworker, and organizational support as moderators of the association between workplace violence exposure and burnout, but did not incorporate post-traumatic stress symptoms as a mediator within a conditional process model [19]. Thus, the present study builds on these distinct lines of research by integrating the indirect association through post-traumatic stress symptoms with the moderating roles of perceived social support and resilience within a conditional process framework.

In particular, social support represents an interpersonal and social resource, whereas resilience represents an individual internal resource for adapting to stress and adversity. However, the roles of these two protective resources may differ across the pathways linking workplace violence exposure, post-traumatic stress symptoms, and burnout. Rather than treating social support and resilience simply as general protective factors that mitigate adverse psychological outcomes, it is necessary to examine separately how they moderate the association between workplace violence exposure and post-traumatic stress symptoms and the direct association between workplace violence exposure and burnout. In addition, examining whether the indirect association through post-traumatic stress symptoms varies according to the levels of these protective resources may provide a more specific understanding of their roles. Therefore, this study aimed to examine the indirect association between workplace violence exposure and burnout through post-traumatic stress symptoms among fire-service-based paramedics and to determine whether social support and resilience separately moderate the association between workplace violence exposure and post-traumatic stress symptoms and the direct association between workplace violence exposure and burnout. We hypothesized positive associations along the workplace violence exposure–post-traumatic stress symptoms–burnout pathway and that higher levels of social support and resilience would separately attenuate the associations of workplace violence exposure with post-traumatic stress symptoms and burnout, resulting in a conditional indirect association through post-traumatic stress symptoms. By examining the moderating roles of social support and resilience in separate conditional process models, this study sought to provide evidence for strategies aimed at strengthening protective resources to address the psychological burden and burnout associated with workplace violence exposure among paramedics.

### 1.2. Purpose

The purpose of this study was to examine the statistical indirect association between workplace violence exposure and burnout through post-traumatic stress symptoms among Korean fire-service-based paramedics and to examine whether social support and resilience separately moderated the association between workplace violence exposure and post-traumatic stress symptoms and the direct association between workplace violence exposure and burnout.

## 2. Materials and Methods

### 2.1. Study Design

This study employed an analytical cross-sectional design to examine the statistical indirect association through post-traumatic stress symptoms and the moderating roles of social support and resilience in the association between workplace violence exposure and burnout among Korean fire-service-based paramedics. The reporting of this study followed the Strengthening the Reporting of Observational Studies in Epidemiology (STROBE) guideline for cross-sectional studies.

### 2.2. Participants and Data Collection

This study was conducted among fire-service-based paramedics who had been working for at least six months at fire stations affiliated with the Busan Metropolitan City Fire and Disaster Headquarters. Participants were eligible if they understood the purpose and procedures of the study and voluntarily agreed to participate.

In South Korea, 119 ambulance personnel are firefighters with professional backgrounds that include medical personnel and Level 1 or Level 2 emergency medical technicians (EMTs) [20]. Level 1 EMTs may qualify through designated university or college programs in emergency medical technology, whereas Level 2 EMTs may qualify through designated training institutions; both require passing a national examination and obtaining official certification [21]. Their authorized scope of prehospital emergency care varies by qualification and includes basic emergency care as well as, for appropriately qualified personnel, advanced procedures such as airway management, intravenous access, and specified medication administration [21].

The required sample size was estimated using G*Power version 3.1 based on a fixed multiple regression model with R^2^ deviation from zero. Assuming α = 0.05, an effect size of f^2^ = 0.15, power = 0.95, and eight predictors, corresponding to the maximum number of predictors in the regression equations, the minimum required sample size was 160. Because this calculation was based on the overall R^2^ rather than the incremental contribution of an interaction term, an additional sensitivity analysis was conducted using a fixed multiple regression model testing R^2^ increase. With the final analytic sample of N = 210, α = 0.05, power = 0.80, one tested predictor, and eight total predictors, the minimum detectable interaction effect was approximately f^2^ = 0.038. Allowing for an anticipated 10% rate of incomplete or unusable responses, the recruitment target was set above the minimum required sample size. Participants were recruited using convenience sampling through an open online invitation to eligible fire-service-based paramedics. According to the most recent official statistics available prior to data collection, 684 fire-service-based paramedics were employed in Busan as of 31 December 2025 [22]. Because the number of eligible individuals who received or viewed the recruitment notice could not be tracked, an exact response rate could not be calculated. A total of 213 individuals responded to the online survey. Although the age item was mandatory, participants entered their age directly rather than selecting from predefined categories. Two respondents entered implausible values that could not be interpreted as valid ages; these responses were excluded, leaving 211 participants for the initial analyses. During subsequent influence diagnostics, one observation was excluded because of disproportionate influence on an interaction term, resulting in a final analytic sample of 210 participants.

Data were collected from 12–13 June 2026. The dataset used in the present study was independent of those used in References [18,19], with no overlap in participants or data. With the cooperation of the Busan Metropolitan City Fire and Disaster Headquarters, the survey URL and QR code were distributed through an internal online bulletin board and the KakaoTalk channel operated by the psychological support team of the Busan Metropolitan City Fire and Disaster Headquarters. Participants accessed the online survey using the provided URL or QR code and were allowed to proceed with the survey only after reviewing the participant information sheet and voluntarily providing informed consent. The survey was administered using Naver Form, and all required items had to be completed before participants could proceed to the next section. The duplicate-response prevention setting in Naver Form was enabled so that each participant could submit the survey only once.

Fire station and operational team identifiers were not collected; therefore, the number of participating fire stations and potential clustering within stations or teams could not be determined from the analytic dataset.

### 2.3. Measures

The online questionnaire consisted of items assessing participants’ general characteristics, including sex, age, marital status, rank, professional qualification, length of service, number of emergency dispatches, and average monthly salary, as well as measures of workplace violence exposure, post-traumatic stress symptoms, burnout, social support, and resilience. Consistent with the scoring procedures described in the original or Korean-validated versions of the instruments, composite total scores were used in the present analyses to represent the overall level of each construct. Where instruments comprised multiple dimensions or subscales, these dimensions were treated as components of the broader construct rather than analyzed separately because the primary aim of this study was to examine the overall constructs within the conditional process models.

#### 2.3.1. Workplace Violence Exposure

Workplace violence exposure was assessed using an instrument originally used by Jung [23] among emergency department nurses and subsequently modified and adapted by Kim [17] to reflect the occupational characteristics of paramedics. The instrument consists of 16 items: four assessing verbal violence, five assessing physical threats, and seven assessing physical violence. The items assess the frequency of workplace violence exposure using five response categories, with the frequency frame differing across the three subscales. For verbal violence, the response options were no experience (0), once per week (1), twice per week (2), three times per week (3), and four or more times per week (4). For physical threats, the response options were no experience (0), once per month (1), twice per month (2), three times per month (3), and four or more times per month (4). For physical violence, the response options were no experience (0), once per year (1), twice per year (2), three times per year (3), and four or more times per year (4). The total score was calculated according to the scoring approach used for the adapted instrument [17], despite the differing recall periods across the three types of workplace violence exposure. Accordingly, the total score should be interpreted as an aggregate indicator of workplace violence exposure rather than as exposure occurring within a single uniform recall period.

#### 2.3.2. Post-Traumatic Stress Symptoms

Post-traumatic stress symptoms were assessed using the Korean version of the PTSD Checklist for DSM-5 (PCL-5), originally developed by Weathers et al. [24] and translated and validated into Korean by Lee et al. [25]. The scale consists of 20 items across four symptom domains: re-experiencing (5 items), avoidance (2 items), negative alterations in cognition and mood (7 items), and hyperarousal (6 items). Participants rated the extent to which they had experienced each symptom during the previous month on a 5-point Likert scale ranging from 0 (“not at all”) to 4 (“extremely”). Total scores range from 0 to 80, with higher scores indicating greater levels of post-traumatic stress symptoms. Cronbach’s α was 0.94 in the original development study and 0.968 in the present study. Consistent with the validated scoring approach, the total score across the 20 items was used as an overall measure of post-traumatic stress symptom severity.

#### 2.3.3. Burnout

Burnout was assessed using the Korean version of the Burnout Assessment Tool (K-BAT), originally developed by Schaufeli et al. [26] and translated and validated into Korean by Cho [27]. The K-BAT consists of 22 items across four dimensions: exhaustion (8 items), mental distance (4 items), impaired cognitive control (5 items), and impaired emotional control (5 items). During the Korean validation process, one Mental Distance item from the original 23-item BAT was removed [27]. Each item is rated on a 5-point Likert scale ranging from 1 (“never”) to 5 (“always”). In the present study, the 22 item scores were summed to calculate a total burnout score, yielding a possible range of 22–110, with higher scores indicating greater burnout. Cronbach’s α was 0.91 in the study by Cho [27] and 0.971 in the present study.

#### 2.3.4. Social Support

Social support was assessed using the Multidimensional Scale of Perceived Social Support (MSPSS), originally developed by Zimet et al. [10] and translated into Korean and applied by Shin and Lee [28].

The original MSPSS consists of 12 items across three subscales assessing perceived social support from family, friends, and significant others, with each item rated on a 7-point Likert scale [10]. In the present study, the Korean version adapted by Shin and Lee [28], which uses a 5-point Likert scale, was employed. This version also consists of 12 items across three subscales assessing perceived support from family, friends, and significant others. Each item is rated from 1 (“strongly disagree”) to 5 (“strongly agree”). Total scores range from 12 to 60, with higher scores indicating greater perceived social support. Cronbach’s α was 0.89 in the study by Shin and Lee [28] and 0.966 in the present study. Although the MSPSS comprises three subscales representing support from family, friends, and significant others, the total score was used in the present study as an overall measure of perceived social support, consistent with the scoring approach used in the Korean version [28].

#### 2.3.5. Resilience

Resilience was assessed using the Korean Resilience Quotient-27 (KRQ-27), developed by Shin, Kim, and Kim [29]. The instrument consists of 27 items, each rated on a 5-point Likert scale ranging from 1 (“strongly disagree”) to 5 (“strongly agree”). Total scores range from 27 to 135, with higher scores indicating greater resilience. Cronbach’s α was 0.89 for the original instrument and 0.896 in the present study. Although the KRQ-27 was originally developed and validated in a younger Korean population, it has subsequently been used in studies of adult Korean firefighters [30,31]. In these studies, the 27-item scale was used to assess resilience among adult firefighters, providing supporting evidence for its use in adult fire-service populations. However, psychometric evidence specifically establishing its factorial validity in adult fire-service-based paramedics remains limited.

### 2.4. Data Analysis

PROCESS Model 8, a regression-based conditional process analysis using ordinary least squares estimation rather than structural equation modeling, was used to examine the conditional indirect association between workplace violence exposure and burnout through post-traumatic stress symptoms, with social support and resilience tested as moderators in separate models. Analyses were conducted using IBM SPSS Statistics version 30 and Hayes’ PROCESS macro version 5.0. Differences in the major study variables according to participants’ general characteristics were examined using independent-samples t-tests or one-way analysis of variance, as appropriate, with Scheffé’s test used for post hoc comparisons. Continuous variables involved in the interaction terms were mean-centered prior to constructing the interaction terms. Accordingly, a value of zero for each centered variable represented its sample mean, allowing the lower-order coefficients to be interpreted at the mean level of the interacting variable. Conditional effects were examined at low, mean, and high levels of the moderators. Statistically supported interactions were further examined using simple slope analyses. Regression coefficients, standard errors, 95% confidence intervals, and changes in R^2^ attributable to the interaction terms were reported. Conditional indirect effects were estimated using bootstrap confidence intervals. Covariates were selected based on their theoretical and methodological relevance to post-traumatic stress symptoms and burnout among fire-service personnel. Given the overlap between rank and length of service, sex, length of service, number of emergency dispatches, and monthly salary were included as covariates because these variables represent demographic and occupational characteristics potentially related to post-traumatic stress symptoms and burnout among fire-service personnel.

Regression assumptions and diagnostics were examined before interpreting the conditional process models. Linearity and homoscedasticity were assessed through examination of residual plots, and the distribution of residuals was evaluated. Multicollinearity was assessed using tolerance and variance inflation factor (VIF) values, and potentially influential observations and outliers were examined using Cook’s distance, leverage, and DFBETA. Following the initial analyses, additional influence diagnostics were conducted in response to concerns raised during peer review. One observation showed a standardized DFBETA of 1.28 for the workplace violence exposure × social support interaction in the model predicting post-traumatic stress symptoms, exceeding the applied absolute cut-off of 1. The exclusion of this observation was not prespecified and was undertaken during the post-review diagnostic assessment. The final analyses were conducted using the remaining 210 participants. Because the Breusch–Pagan test indicated heteroskedasticity in the mediator models, HC3 heteroskedasticity-consistent standard errors were used for the regression estimates to provide inference robust to heteroskedasticity. As an additional sensitivity analysis, each moderation model was estimated with the other moderator included as a covariate to assess whether the interaction remained supported after accounting for the correlation between social support and resilience. The statistical significance of the indirect effects and moderated mediation effects was determined based on 95% confidence intervals derived from 5000 bootstrap samples. To account for multiple testing, the Holm correction was applied to the four interaction tests: workplace violence exposure × social support and workplace violence exposure × resilience, each examined in the models predicting post-traumatic stress symptoms and burnout.

### 2.5. Ethical Considerations

This study was conducted after obtaining approval from the Institutional Review Board (IRB) of Dong-A University (Approval No. 2-1040709-AB-*n*-01-202604-HR-017-04; 19 May 2026). The participant information sheet described the purpose and procedures of the study, the voluntary nature of participation, participants’ right to refuse or withdraw from the study at any time without penalty, and the potential benefits and risks of participation. The collected data were used solely for research purposes, and participants’ anonymity and confidentiality were maintained by not collecting personally identifiable information or by managing such information separately.

Participants were informed that the collected data would be retained for three years after completion of the study and subsequently permanently destroyed. Only participants who reviewed the participant information sheet and voluntarily provided informed consent were permitted to complete the survey. Upon completion of the survey, participants received a small token of appreciation for their participation.

## 3. Results

### 3.1. General Characteristics of the Participants

The general characteristics of the 210 participants are presented in Table 1. Of the participants, 166 (79.0%) were men and 44 (21.0%) were women. The mean age was 35.10 ± 5.20 years. The largest age group was 31–35 years (*n* = 74, 35.2%), followed by ≤30 years (*n* = 49, 23.3%), 36–40 years (*n* = 47, 22.4%), and ≥41 years (*n* = 40, 19.0%).

Regarding marital status, 121 participants (57.6%) were married, 81 (38.6%) were unmarried, and 8 (3.8%) reported other marital statuses. Regarding rank, 80 participants (38.1%) were senior firefighters, 66 (31.4%) were firefighters, and 64 (30.5%) were fire sergeants or higher. In terms of professional qualifications, registered nurses accounted for the largest proportion (*n* = 136, 64.8%), followed by Level 1 emergency medical technicians (*n* = 33, 15.7%), Level 2 emergency medical technicians (*n* = 29, 13.8%), and other qualifications (*n* = 12, 5.7%).

The mean length of service was 6.26 ± 4.75 years, with the largest proportion of participants having ≥1 to <5 years of service (*n* = 84, 40.0%). The mean number of emergency dispatches per day was 7.41 ± 2.78, with 143 participants (68.1%) reporting ≥5 to <10 dispatches per day. The mean monthly salary was KRW 3.68 ± 0.51 million, with 78 participants (37.1%) earning ≥KRW 4.00 million, 67 (31.9%) earning <KRW 3.50 million, and 65 (31.0%) earning ≥KRW 3.50 to <4.00 million (Table 1).

In the final analytic sample of 210 participants, differences in the major study variables according to participants’ general characteristics were also examined. Workplace violence exposure differed significantly according to the number of emergency dispatches, whereas post-traumatic stress symptoms differed significantly according to rank and length of service. Burnout differed significantly according to sex, rank, length of service, number of emergency dispatches, and monthly salary. Social support differed significantly according to age and monthly salary, whereas resilience differed significantly according to sex, length of service, and monthly salary. Detailed results of these comparisons, including category-specific sample sizes, test statistics and *p*-values, are presented in Supplementary Table S1.

### 3.2. Descriptive Statistics and Correlations Among the Study Variables

Descriptive statistics for the main variables showed that the mean score for workplace violence exposure was 11.19 ± 8.42. By subdomain, the mean scores were 4.92 ± 3.06 for verbal violence, 4.44 ± 3.74 for physical threats, and 1.83 ± 2.85 for physical violence. The mean scores were 16.94 ± 14.66 for post-traumatic stress symptoms, 48.10 ± 16.94 for burnout, 47.68 ± 9.75 for social support, and 88.45 ± 12.36 for resilience. Cronbach’s α values for the measurement instruments used in this study ranged from 0.782 to 0.971.

Correlation analysis among the main variables showed that burnout was significantly positively correlated with workplace violence exposure (r = 0.345, *p* < 0.001) and post-traumatic stress symptoms (r = 0.740, *p* < 0.001). In contrast, burnout was significantly negatively correlated with social support (r = −0.285, *p* < 0.001) and resilience (r = −0.385, *p* < 0.001).

Workplace violence exposure was significantly positively correlated with post-traumatic stress symptoms (r = 0.380, *p* < 0.001), but was not significantly correlated with social support (r = −0.087) or resilience (r = −0.004). Post-traumatic stress symptoms were significantly negatively correlated with social support (r = −0.301, *p* < 0.001) and resilience (r = −0.298, *p* < 0.001). Social support and resilience were significantly positively correlated (r = 0.467, *p* < 0.001) (Table 2).

### 3.3. Moderation and Conditional Effects Analyses

**Figure 1:**
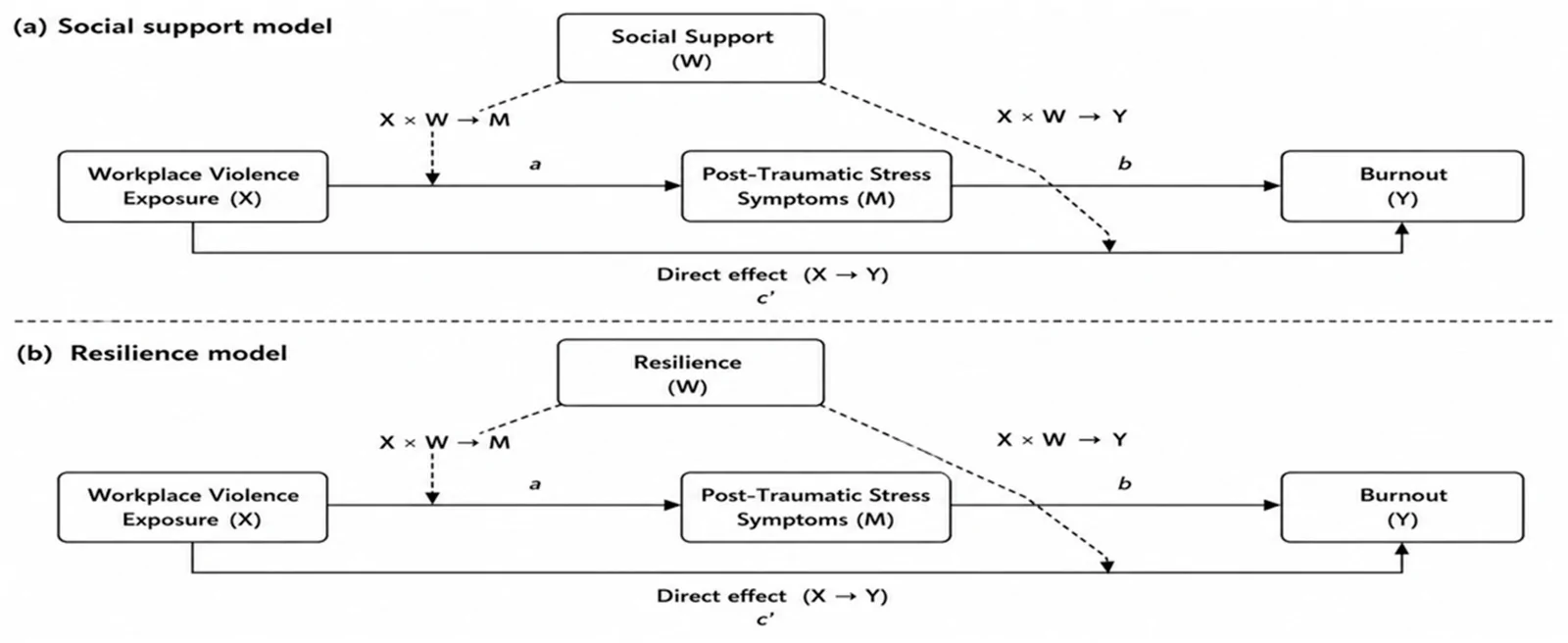
Conceptual models of the moderated mediation analyses (PROCESS Model 8). (**a**) Social support model; (**b**) resilience model. X = workplace violence exposure; M = post-traumatic stress symptoms; Y = burnout; W = moderator. The labels a, b, and c′ represent the X → M, M → Y, and direct X → Y paths, respectively. Solid arrows indicate the regression paths, whereas dashed arrows indicate moderation of the X → M and X → Y paths. Social support and resilience were examined in separate models.

PROCESS Model 8 was used to examine the conditional indirect association between workplace violence exposure and burnout through post-traumatic stress symptoms, with social support and resilience tested as moderators in separate models. Social support and resilience were analyzed using separate PROCESS Model 8 analyses, and the two moderators were not simultaneously included in the same model. Sex, length of service, number of emergency dispatches, and monthly salary were included as covariates in the analyses. The research models used in the analyses are presented in Figure 1.

Diagnostic assessment indicated no substantial multicollinearity, with VIF values ranging from approximately 1.06 to 1.73 across the fitted models. The Breusch–Pagan test indicated heteroskedasticity in the mediator models; therefore, HC3 heteroskedasticity-consistent standard errors were used as the primary standard errors for regression inference. Influence diagnostics were examined using Cook’s distance, leverage, and DFBETA. The maximum Cook’s distance was approximately 0.20, indicating no observations of concern based on Cook’s distance. Approximately six observations exceeded the leverage criterion; however, these observations were retained because their leverage was primarily attributable to covariate values and was not considered sufficiently influential to warrant exclusion. One observation showed disproportionate influence on the interaction term based on DFBETA and was excluded from the final analyses. Accordingly, the final analytic sample consisted of 210 participants. All conditional process analyses reported below were based on this final sample and used HC3 heteroskedasticity-consistent standard errors for regression inference.

#### 3.3.1. Moderating Effects of Social Support and Resilience on the Association Between Workplace Violence Exposure and Post-Traumatic Stress Symptoms

The interaction between workplace violence exposure and social support was statistically significant for post-traumatic stress symptoms (B = −0.033, HC3 SE = 0.010, 95% CI [−0.053, −0.013], *p* = 0.002), with the interaction term accounting for an additional 3.6% of the variance (ΔR^2^ = 0.036). In contrast, the interaction between workplace violence exposure and resilience was not statistically significant (B = −0.013, HC3 SE = 0.011, 95% CI [−0.036, 0.009], *p* = 0.245; ΔR^2^ = 0.008) (Table 3).

In sensitivity analyses additionally adjusting for the other moderator, the workplace violence exposure × social support interaction remained statistically significant for post-traumatic stress symptoms after adjustment for resilience (B = −0.031, HC3 SE = 0.011, 95% CI [−0.053, −0.010], *p* = 0.004), whereas the workplace violence exposure × resilience interaction remained non-significant after adjustment for social support (B = −0.012, HC3 SE = 0.013, 95% CI [−0.037, 0.014], *p* = 0.365).

#### 3.3.2. Moderating Effects of Social Support and Resilience on the Associations of Workplace Violence Exposure and Post-Traumatic Stress Symptoms with Burnout

Post-traumatic stress symptoms were significantly positively associated with burnout in both the social support model (B = 0.731, HC3 SE = 0.072, 95% CI [0.589, 0.874], *p* <0.001) and the resilience model (B = 0.705, HC3 SE = 0.071, 95% CI [0.565, 0.844], *p* < 0.001). The interaction between workplace violence exposure and social support was statistically significant (B = −0.022, HC3 SE = 0.009, 95% CI [−0.040, −0.004], *p* = 0.015), whereas the interaction between workplace violence exposure and resilience was nominally significant (B = −0.023, HC3 SE = 0.011, 95% CI [−0.046, 0.000], *p* = 0.047) (Table 4).

In sensitivity analyses, after also adjusting for the other moderator, the workplace violence exposure × social support interaction remained statistically significant for burnout after adjustment for resilience (B = −0.022, HC3 SE = 0.010, 95% CI [−0.042, −0.002], *p* = 0.033). The workplace violence exposure × resilience interaction also remained nominally significant after adjustment for social support (B = −0.024, HC3 SE = 0.011, 95% CI [−0.046, −0.001], *p* = 0.041). Simple slope analyses indicated that the conditional direct association between workplace violence exposure and burnout was statistically significant at low levels of both social support and resilience, but not at the mean or high levels of either moderator (Table 5).

#### 3.3.3. Conditional Direct and Indirect Effects and Indices of Moderated Mediation According to Levels of Social Support and Resilience

For social support, the conditional indirect association of workplace violence exposure with burnout through post-traumatic stress symptoms was statistically significant at low (Effect = 0.639, 95% CI [0.416, 0.905]) and mean (Effect = 0.404, 95% CI [0.205, 0.648]) levels of social support, but not at a high level (Effect = 0.169, 95% CI [−0.113, 0.475]). The conditional direct association between workplace violence exposure and burnout was statistically significant at a low level of social support (Effect = 0.368, 95% CI [0.074, 0.662]), but not at the mean (Effect = 0.150, 95% CI [−0.079, 0.380]) or high (Effect = −0.067, 95% CI [−0.350, 0.216]) levels. The index of moderated mediation was −0.024 (95% CI [−0.041, −0.009]), indicating a statistically significant moderated indirect association.

**Figure 2:**
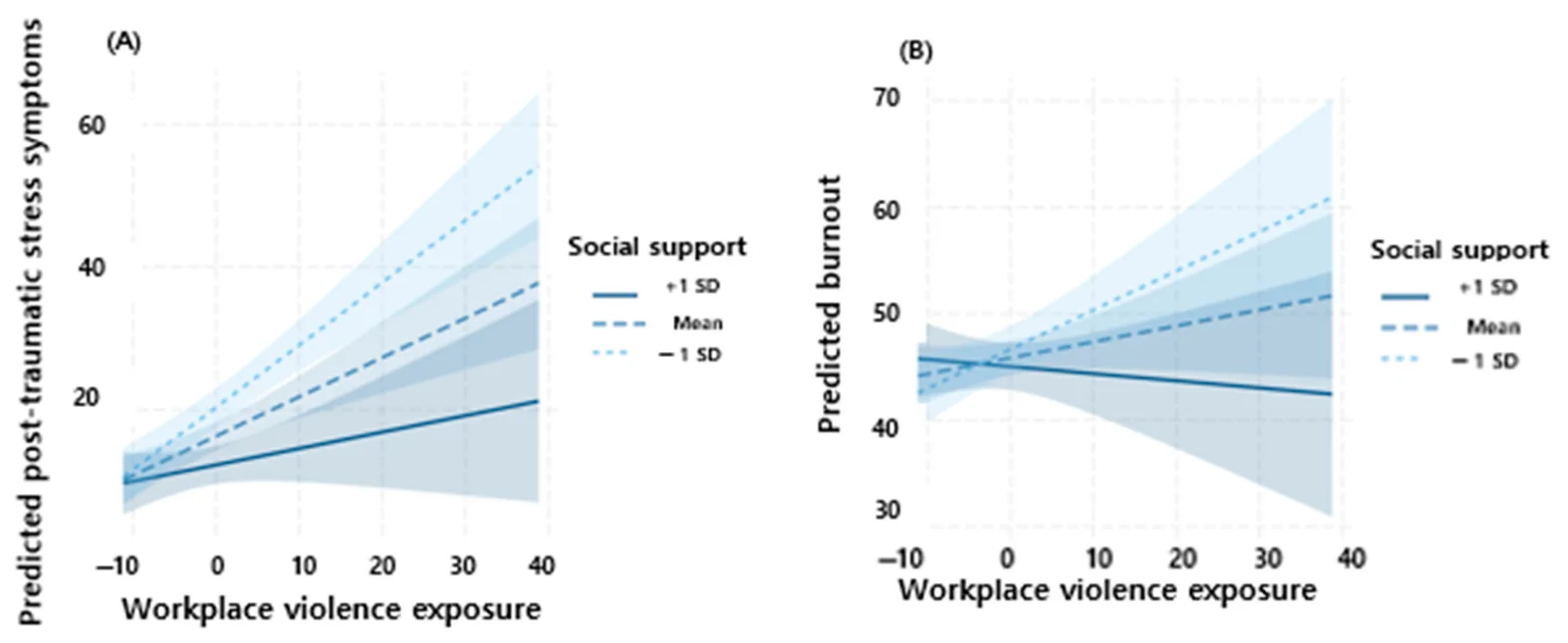
Interaction effects of social support on the associations of workplace violence exposure with post-traumatic stress symptoms and burnout. (**A**) Predicted post-traumatic stress symptoms according to workplace violence exposure at low (−1 SD), mean (0), and high (+1 SD) levels of social support. (**B**) Predicted burnout according to workplace violence exposure at low (−1 SD), mean (0), and high (+1 SD) levels of social support. Workplace violence exposure on the x-axis is mean-centered.

**Figure 3:**
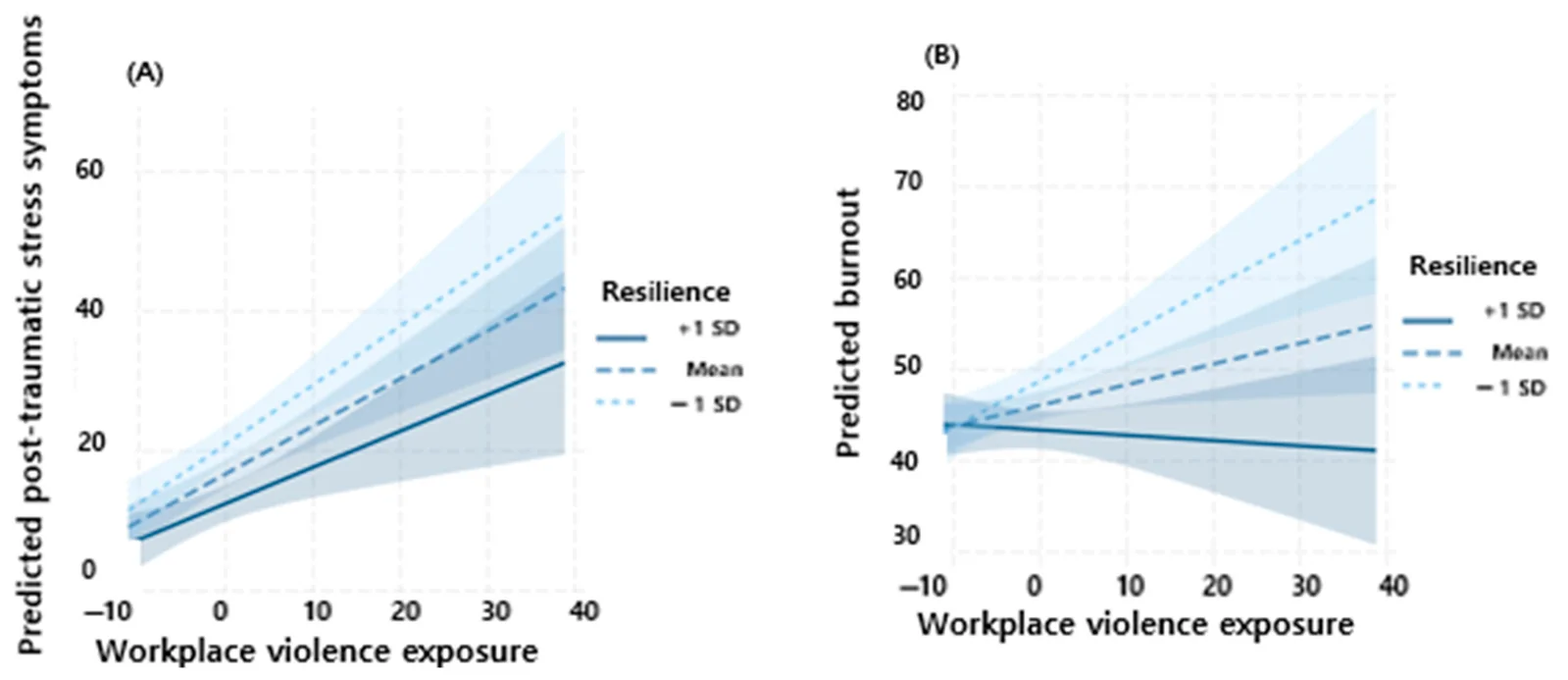
Interaction effects of resilience on the associations of workplace violence exposure with post-traumatic stress symptoms and burnout. (**A**) Predicted post-traumatic stress symptoms according to workplace violence exposure at low (−1 SD), mean (0), and high (+1 SD) levels of resilience. (**B**) Predicted burnout according to workplace violence exposure at low (−1 SD), mean (0), and high (+1 SD) levels of resilience. Workplace violence exposure on the x-axis is mean-centered.

For resilience, the conditional indirect association of workplace violence exposure with burnout through post-traumatic stress symptoms was statistically significant at low (Effect = 0.598, 95% CI [0.288, 0.980]), mean (Effect = 0.482, 95% CI [0.276, 0.725]), and high (Effect = 0.366, 95% CI [0.117, 0.623]) levels of resilience. The conditional direct association between workplace violence exposure and burnout was statistically significant at a low level of resilience (Effect = 0.511, 95% CI [0.105, 0.917]), but not at the mean (Effect = 0.227, 95% CI [−0.011, 0.465]) or high (Effect = −0.058, 95% CI [−0.382, 0.266]) levels. The index of moderated mediation was −0.009 (95% CI [−0.027, 0.006]) and was not statistically significant (Table 5). The interaction patterns for social support and resilience are illustrated in Figure 2 and Figure 3, respectively.

## 4. Discussion

### 4.1. Principal Findings

This study examined the statistical indirect association between workplace violence exposure and burnout through post-traumatic stress symptoms and the moderating roles of social support and resilience among fire-service-based paramedics. Workplace violence exposure was positively associated with post-traumatic stress symptoms, which in turn were positively associated with burnout. A significant statistical indirect association through post-traumatic stress symptoms was observed. Social support moderated the associations of workplace violence exposure with both post-traumatic stress symptoms and burnout, and the index of moderated mediation for social support was significant. In contrast, evidence for resilience as a moderator was less robust, and its index of moderated mediation was not statistically significant.

### 4.2. Workplace Violence, Post-Traumatic Stress Symptoms, and Burnout

Workplace violence exposure among fire-service-based paramedics was significantly associated with post-traumatic stress symptoms. This finding is consistent with the study by Hoell et al. [32], which reported that repeated exposure to work-related traumatic events was associated with post-traumatic stress symptoms among paramedics, and with the study by McGuire et al. [33], which reported that workplace violence among EMS personnel was associated with psychological health and trauma-related responses. Violence experienced by paramedics may be associated with trauma-related psychological burden because it can be perceived as a physical and psychological threat in field settings that are difficult to predict and control. Therefore, workplace violence should be recognized as an occupational risk factor distinct from general job stress, and post-traumatic stress symptoms among paramedics exposed to violence should be assessed and appropriately addressed.

Post-traumatic stress symptoms were significantly positively associated with burnout among paramedics. This may be explained by the possibility that the persistent emotional and cognitive burden accompanying post-traumatic stress symptoms, such as re-experiencing, hyperarousal, and avoidance, is associated with the depletion of psychological resources required for job performance and may thereby be linked to burnout.

This finding is consistent with the study by Mitani et al. [6], which examined the effect of post-traumatic stress symptoms on burnout among fire service workers. Therefore, prevention and management of burnout among paramedics should consider not only general occupational stressors, such as shift work and workload, but also post-traumatic stress symptoms. Female sex was also associated with approximately 9–10 points higher burnout scores in both models after adjustment for the other variables included in the models. Given the smaller number of female participants and the cross-sectional design, this finding should be interpreted cautiously and warrants further investigation of potential sex differences in burnout among fire-service-based paramedics.

Taken together, these findings indicated a statistical indirect association between workplace violence exposure and burnout through post-traumatic stress symptoms. However, because the data were cross-sectional, this pattern should not be interpreted as evidence that workplace violence temporally preceded post-traumatic stress symptoms or that post-traumatic stress symptoms causally contributed to subsequent burnout.

The statistical indirect association through post-traumatic stress symptoms should therefore be considered when understanding the relationship between workplace violence exposure and burnout. Furthermore, considering the findings of a meta-analysis showing that psychological interventions for emergency response personnel are effective in reducing post-traumatic stress symptoms [34], the management of burnout among paramedics should consider both organizational efforts to reduce exposure to violence and the assessment of post-traumatic stress symptoms and provision of evidence-based psychological support.

### 4.3. Moderating Roles of Social Support and Resilience

This study separately examined the moderating effects of social support and resilience. In the final analytic sample (N = 210), social support significantly moderated the associations of workplace violence exposure with both post-traumatic stress symptoms and burnout. These interactions remained statistically significant after Holm correction for multiple testing. However, the local effect size of the interaction between workplace violence exposure and social support for burnout (f^2^ = 0.034) was slightly below the approximately f^2^ = 0.038 minimum detectable effect identified in the sensitivity analysis; thus, the magnitude and precision of this interaction should be interpreted cautiously despite its statistical significance.

These findings should not be interpreted as demonstrating a statistical difference between the moderating roles of social support and resilience, because the moderators were examined in separate models and their interaction coefficients were not directly compared. Social support measured in this study refers to perceived support from family, friends, and significant others, and the moderating associations identified in this study are consistent with the stress-buffering hypothesis proposed by Cohen and Wills [11], although the cross-sectional design precludes causal interpretation of social support as a buffering mechanism. Accordingly, the present findings should not be interpreted as direct evidence of a buffering effect of organizational or coworker support. Future studies should distinguish perceived social support from work-related support and examine their respective roles.

Resilience has been described as an individual internal resource for adapting to stress and adversity [12,13], and previous research has reported that higher resilience is associated with a lower prevalence of post-traumatic stress disorder and burnout [14]. In the present study, the interaction between workplace violence exposure and resilience was not statistically significant for post-traumatic stress symptoms. For burnout, the interaction was nominally significant (*p* = 0.047) but did not remain statistically significant after correction for multiple testing (Holm-adjusted *p* = 0.094). Thus, the evidence supporting resilience as a moderator was less robust. In the final analytic sample, both moderators showed negative interaction estimates for burnout, whereas the main difference in their observed moderation patterns was evident in the pathway from workplace violence exposure to post-traumatic stress symptoms. This comparison is descriptive and does not establish a statistically significant difference between the moderators.

In the final analytic sample, the index of moderated mediation was statistically significant for social support (index = −0.024, 95% bootstrap CI [−0.041, −0.009]), indicating that the indirect association between workplace violence exposure and burnout through post-traumatic stress symptoms was conditional on the level of social support. In contrast, the index of moderated mediation for resilience was not statistically significant (index = −0.009, 95% bootstrap CI [−0.027, 0.006]). Thus, although the conditional indirect association through post-traumatic stress symptoms was statistically significant at all examined levels of resilience, this alone does not indicate that the magnitude of the indirect association varied significantly across levels of resilience.

Importantly, the sensitivity analysis indicated that the statistical significance of the workplace violence exposure × social support interaction predicting post-traumatic stress symptoms depended on the exclusion of one influential observation (*p* = 0.211 for N = 211 versus *p* = 0.002 for N = 210). Similarly, the index of moderated mediation for social support was not statistically significant in the initial sample of 211 participants. In contrast, the interaction between workplace violence exposure and social support in predicting burnout remained statistically significant in both samples. Therefore, the findings concerning social support moderation of the post-traumatic stress symptoms pathway and the corresponding moderated indirect association should be interpreted cautiously.

### 4.4. Comparison with Previous Studies of Korean Paramedics

The present study builds conceptually and analytically on previous research among Korean paramedics by integrating post-traumatic stress symptoms, perceived social support, and resilience within a conditional process framework. Unlike previous studies that examined resilience within a path model [18] or specific sources of social support as moderators of the workplace violence–burnout association [19], the present study examined the statistical indirect association through post-traumatic stress symptoms together with the moderating roles of perceived social support and resilience.

A comparable directional pattern was reported by Sung and Lee [18], who found positive standardized paths from workplace violence exposure to post-traumatic stress symptoms (β = 0.39) and from post-traumatic stress symptoms to burnout (β = 0.52), together with a significant indirect association of workplace violence exposure with burnout through post-traumatic stress symptoms (β = 0.23). Although these effect estimates are not directly comparable with the unstandardized coefficients in the present study because of differences in measures and analytic approaches, both studies showed the same directional pattern linking workplace violence exposure, post-traumatic stress symptoms, and burnout.

Among the support sources examined by Lee and Lee [19], family support is conceptually the closest counterpart to the perceived social support assessed in the present study. Lee and Lee [19] found that family support significantly moderated the association between workplace violence exposure and burnout (β = −0.249, *p* = 0.008), with the positive association becoming weaker as family support increased; the association was significant at low and mean levels of family support but not at a high level. This directional pattern is consistent with the present finding that higher perceived social support attenuated the association between workplace violence exposure and burnout. However, the magnitude of the interaction coefficients should not be directly compared because the studies used different social support measures, coefficient metrics, and analytic models.

Taken together, these comparisons suggest convergence in the directional patterns observed across studies of Korean paramedics, while differences in study design, measurement, and analytic approach should be considered when comparing the magnitude of the reported associations.

### 4.5. Implications for Practice and Policy

Based on the findings of this study, both organizational responses to workplace violence exposure and the strengthening of protective resources may be considered as part of efforts to address burnout and promote mental health among paramedics. First, incidents of violence should be recognized not as occupational problems that individuals are expected to endure, but as occupational risk factors that should be managed at the organizational level. Reporting and response procedures for violent incidents and support systems for paramedics affected by violence should be strengthened. In particular, it is important to ensure access to psychological counseling and professional mental health services following exposure to violence.

Second, considering the characteristics of the protective resources examined in this study, efforts to strengthen perceived support from personal social networks, including family, friends, and significant others, as well as individual resilience resources, may be beneficial. At the organizational level, fire-service agencies may also consider broader strategies that facilitate access to interpersonal and psychosocial support; however, organizational support itself was not directly measured in the present study. Rather than approaching resilience solely as an individual responsibility, organizational environments that ensure adequate rest, workload management, and psychological safety should also be considered.

### 4.6. Strengths and Limitations

This study has several strengths. It examined the statistical indirect association between workplace violence exposure and burnout through post-traumatic stress symptoms while assessing the moderating roles of perceived social support and resilience in separate conditional process models. In addition, the study used validated measures and robustness procedures, including HC3 heteroskedasticity-consistent standard errors, influence diagnostics, sensitivity analyses, and adjustment for multiple testing.

This study also has several limitations. First, because this study used a cross-sectional design, the temporal sequence and causal relationships among workplace violence exposure, post-traumatic stress symptoms, burnout, social support, and resilience cannot be established. In particular, the reverse ordering, in which burnout contributes to post-traumatic stress symptoms, is also consistent with the cross-sectional data and cannot be ruled out. Therefore, the indirect and moderating associations identified in this study should not be interpreted as evidence of causal effects. Longitudinal studies are needed to clarify the temporal relationships among these variables.

Second, participants were recruited from fire stations affiliated with a single metropolitan fire and disaster headquarters in Busan, South Korea, which may limit the generalizability of the findings to paramedics working in other regions or emergency medical service systems. In addition, 64.8% of participants were registered nurses, and this training composition may further limit generalizability to EMS systems with different professional or training mixes. Future studies should include participants from multiple regions and organizations. In addition, because station- and team-level identifiers were not collected, potential clustering of participants within fire stations or operational teams could not be evaluated or accounted for using cluster-robust or multilevel methods. Future studies should therefore collect organizational identifiers to assess and appropriately account for such within-group dependence. Finally, the sensitivity analysis indicated that the present sample may have had insufficient statistical power to detect interaction effects smaller than approximately f^2^ = 0.038.

Third, all study variables were assessed using self-report measures, which may be subject to recall bias and common method bias. The workplace violence exposure score also combined items assessed over different recall periods, which may limit the interpretability of the aggregate score. In addition, although the KRQ-27 has been used in adult firefighter populations, further psychometric evaluation, including examination of its factorial validity, is warranted specifically among adult fire-service-based paramedics. Although post-traumatic stress symptoms and burnout are conceptually distinct, they may share some psychological and functional manifestations. Post-traumatic stress symptoms are trauma-related responses characterized by symptoms such as intrusion, avoidance, negative alterations in cognition and mood, and hyperarousal [24,25], whereas burnout reflects a work-related state characterized by exhaustion and psychological distancing from work [26,27]. Nevertheless, both constructs may involve emotional distress, cognitive difficulties, and impaired occupational functioning, which may partly contribute to the strong association observed in this study. This overlap in psychological manifestations and item content may have inflated the estimated b path and, consequently, the indirect association; however, the extent of any such inflation cannot be determined from the present data. Future research incorporating multiple data sources or objective measures, where feasible, may help address these measurement-related limitations.

Finally, participation in the online survey was voluntary, which may have introduced selection bias because individuals who chose to participate may have differed systematically from those who did not. These limitations should be considered when interpreting the findings of this study.

## 5. Conclusions

In this study, workplace violence exposure among fire-service-based paramedics showed a significant indirect association with burnout through post-traumatic stress symptoms. In the final analytic sample (N = 210), social support significantly moderated the associations of workplace violence exposure with both post-traumatic stress symptoms and burnout, and its index of moderated mediation was significant. However, the interaction predicting post-traumatic stress symptoms and the index of moderated mediation were not statistically significant in the initial sample (N = 211), indicating sensitivity to the exclusion of one influential observation. The social support interaction predicting burnout remained significant in both samples.

Evidence for resilience as a moderator was less robust after correction for multiple testing, and its index of moderated mediation was not statistically significant. These findings should be interpreted cautiously given the sensitivity of the social support findings to one observation and the cross-sectional design, which precludes causal inference. Further longitudinal studies are needed to verify these associations.

## Figures and Tables

**Table 1:** General characteristics of the participants (N = 210).

Variable	Category	N (%)	M ± SD
Sex	Male	166 (79.0)	
	Female	44 (21.0)	
Age (years)	≤30	49 (23.3)	35.10 ± 5.20
	31–35	74 (35.2)	
	36–40	47 (22.4)	
	≥41	40 (19.0)	
Marital status	Unmarried	81 (38.6)	
	Married	121 (57.6)	
	Other	8 (3.8)	
Rank	Firefighter	66 (31.4)	
	Senior firefighter	80 (38.1)	
	Fire sergeant or higher	64 (30.5)	
Professional qualification	Level 1 emergency medical technician	33 (15.7)	
	Registered nurse	136 (64.8)	
	Level 2 emergency medical technician	29 (13.8)	
	Other	12 (5.7)	
Length of service (years)	<1	18 (8.6)	6.26 ± 4.75
	1–<5	84 (40.0)	
	5–<10	56 (26.7)	
	≥10	52 (24.8)	
Number of dispatches	<5	22 (10.5)	7.41 ± 2.78
	5–<10	143 (68.1)	
	≥10	45 (21.4)	
Monthly salary (10,000 KRW)	<350	67 (31.9)	367.83 ± 51.30
	350–<400	65 (31.0)	
	≥400	78 (37.1)	

*Note:* SD = standard deviation; KRW = Korean won.

**Table 2:** Descriptive statistics, reliability, and correlations among the study variables.

Variable	M ± SD	Observed Range	Possible Range	Cronbach’s α	1	2	3	4	5
1. Workplace violence exposure	11.19 ± 8.42	0–50	0–64	0.908	1				
Verbal violence	4.92 ± 3.06	0–16	0–16	0.852					
Physical threat	4.44 ± 3.74	0–20	0–20	0.841					
Physical violence	1.83 ± 2.85	0–17	0–28	0.782					
2. Post-traumatic stress symptoms	16.94 ± 14.66	0–60	0–80	0.968	0.380 ***	1			
3. Burnout	48.10 ± 16.94	22–103	22–110	0.971	0.345 ***	0.740 ***	1		
4. Social support	47.68 ± 9.75	12–60	12–60	0.966	−0.087	−0.301 ***	−0.285 ***	1	
5. Resilience	88.45 ± 12.36	38–135	27–135	0.896	−0.004	−0.298 ***	−0.385 ***	0.467 ***	1

*Note:* SD = standard deviation. Pearson’s *r* was used for correlation analysis. *** *p* < 0.001.

**Table 3:** Regression models predicting post-traumatic stress symptoms with social support and resilience as moderators.

Predictor	B	SE (HC3)	95% CI	t	*p*	f^2^
**A. Social Support**						
Sex (Female)	1.984	2.138	(−2.231, 6.199)	0.928	0.354	
Length of service	0.161	0.253	(−0.338, 0.661)	0.637	0.525	
Number of emergency dispatches	−0.289	0.406	(−1.090, 0.511)	−0.712	0.477	
Monthly salary	0.008	0.023	(−0.037, 0.054)	0.360	0.719	
Workplace violence exposure	0.552	0.140	(0.275, 0.829)	3.935	<0.001	
Social support	−0.415	0.091	(−0.595, −0.235)	−4.545	<0.001	
Workplace violence exposure × Social support	−0.033	0.010	(−0.053, −0.013)	−3.218	0.002	0.049
R^2^	0.267					
Adjusted R^2^	0.242					
F (HC3)	12.349					
*p*	<0.001					
ΔR^2^	0.036					
ΔF (HC3)	10.355					
ΔF (*p*)	0.002					
**B. Resilience**						
Sex (Female)	0.946	2.153	(−3.300, 5.192)	0.439	0.661	
Length of service	0.218	0.234	(−0.243, 0.680)	0.932	0.352	
Number of emergency dispatches	−0.391	0.449	(−1.276, 0.494)	−0.871	0.385	
Monthly salary	0.010	0.021	(−0.031, 0.051)	0.478	0.633	
Workplace violence exposure	0.684	0.148	(0.391, 0.976)	4.611	<0.001	
Resilience	−0.344	0.083	(−0.508, −0.180)	−4.131	<0.001	
Workplace violence exposure × Resilience	−0.013	0.011	(−0.036, 0.009)	−1.165	0.245	0.011
R^2^	0.253					
Adjusted R^2^	0.227					
F (HC3)	6.944					
*p*	<0.001					
ΔR^2^	0.008					
ΔF (HC3)	1.358					
ΔF (*p*)	0.245					

*Note:* B = unstandardized regression coefficient; SE = standard error. Sex was coded with male as the reference category. f^2^ represents the local effect size of the interaction term. The models were adjusted for sex, length of service, number of emergency dispatches, and monthly salary.

**Table 4:** Regression Models Predicting Burnout with Social Support and Resilience as Moderators.

Predictor	B	SE (HC3)	95% CI	*t*	*p*	f^2^
**A. Social Support**						
Sex (Female)	10.009	1.758	(6.543, 13.475)	5.695	<0.001	
Length of service	0.435	0.198	(0.045, 0.825)	2.198	0.029	
Number of emergency dispatches	0.024	0.376	(−0.717, 0.765)	0.064	0.949	
Monthly salary	0.015	0.019	(−0.023, 0.052)	0.772	0.441	
Workplace violence exposure	0.150	0.117	(−0.079, 0.380)	1.291	0.198	
Post-traumatic stress symptoms	0.731	0.072	(0.589, 0.874)	10.130	<0.001	
Social support	−0.083	0.102	(−0.285, 0.118)	−0.817	0.415	
Workplace violence exposure × Social support	−0.022	0.009	(−0.040, −0.004)	−2.453	0.015	0.034
R^2^	0.642					
Adjusted R^2^	0.628					
F (HC3)	70.525					
*p*	<0.001					
ΔR^2^	0.012					
ΔF (HC3)	6.018					
ΔF (*p*)	0.015					
**B. Resilience**						
Sex (Female)	9.460	1.810	(5.890, 13.029)	5.225	<0.001	
Length of service	0.403	0.186	(0.037, 0.770)	2.170	0.031	
Number of emergency dispatches	−0.062	0.328	(−0.708, 0.585)	−0.188	0.851	
Monthly salary	0.017	0.018	(−0.019, 0.052)	0.912	0.363	
Workplace violence exposure	0.227	0.121	(−0.011, 0.465)	1.877	0.062	
Post-traumatic stress symptoms	0.705	0.071	(0.565, 0.844)	9.956	<0.001	
Resilience	−0.219	0.093	(−0.402, −0.036)	−2.363	0.019	
Workplace violence exposure × Resilience	−0.023	0.011	(−0.046, 0.000)	−2.003	0.047	0.053
R^2^	0.663					
Adjusted R^2^	0.649					
F (HC3)	68.123					
*p*	<0.001					
ΔR^2^	0.018					
ΔF (HC3)	4.011					
ΔF (*p*)	0.047					

*Note:* B = unstandardized regression coefficient; SE = standard error. Sex was coded with male as the reference category. f^2^ represents the local effect size of the interaction term. The models were adjusted for sex, length of service, number of emergency dispatches, and monthly salary.

**Table 5:** Conditional Direct and Indirect Effects of Workplace Violence Exposure on Burnout According to Levels of Social Support and Resilience.

Moderator/Effect	Moderator Level	Effect	SE	95% CI
A. Social Support				
Indirect effect through post-traumatic stress symptoms	−9.747 (Low)	0.639	0.124	[0.416, 0.905]
	0.000 (Mean)	0.404	0.115	[0.205, 0.648]
	9.747 (High)	0.169	0.150	[−0.113, 0.475]
Direct effect	−9.747 (Low)	0.368	0.149	[0.074, 0.662]
	0.000 (Mean)	0.150	0.117	[−0.079, 0.380]
	9.747 (High)	−0.067	0.144	[−0.350, 0.216]
Index of moderated mediation	—	−0.024	0.008	[−0.041, −0.009]
B. Resilience				
Indirect effect through post-traumatic stress symptoms	−12.364 (Low)	0.598	0.174	[0.288, 0.980]
	0.000 (Mean)	0.482	0.115	[0.276, 0.725]
	12.364 (High)	0.366	0.129	[0.117, 0.623]
Direct effect	−12.364 (Low)	0.511	0.206	[0.105, 0.917]
	0.000 (Mean)	0.227	0.121	[−0.011, 0.465]
	12.364 (High)	−0.058	0.164	[−0.382, 0.266]
Index of moderated mediation	—	−0.009	0.008	[−0.027, 0.006]

*Note:* SE = standard error; CI = confidence interval. Conditional indirect effects and indices of moderated mediation are based on bootstrap confidence intervals. Low, mean, and high levels represent the moderator values used in the conditional effect analyses.

## Data Availability

The datasets generated and/or analyzed during the current study are not publicly available due to privacy and ethical restrictions. De-identified data may, however, be made available from the corresponding author upon reasonable request and with Institutional Review Board approval.

## Supplementary Materials

The following supporting information can be downloaded at: https://www.mdpi.com/article/10.3390/healthcare14213607/s1. Table S1. Differences in the major study variables according to participants’ general characteristics (N = 210); Table S2. Sensitivity analyses of interaction effects and indices of moderated mediation in the initial sample (N = 211); and the completed STROBE checklist for cross-sectional studies.
