# Supplementary material for: Workplace Violence Exposure and Burnout Among Fire-Service-Based Paramedics in South Korea: The Indirect Association Through Post-Traumatic Stress Symptoms and the Moderating Roles of Social Support and Resilience
Source: Healthcare (Basel). 2026 Oct 24;14(21):3607. doi: 10.3390/healthcare14213607 (PMC13763096; doi:10.3390/healthcare14213607)
Supplement: Supplementary file 1 [file healthcare-14-03607-s001.zip › healthcare-4552848-supplementary.pdf]

Supplementary Table S1. Differences in the Major Study Variables According to Participants' General Characteristics in the Final Sample (N = 210)

A. Workplace violence exposure

Characteristic	Category	n	M ± SD	t/F	p	Scheffé
Sex	Male	166	11.44 ± 8.18	0.833	.406	
	Female	44	10.25 ± 9.30			
Age (years)	≤30	49	9.14 ± 6.55	2.593	.054	
	31–35	74	12.70 ± 9.69			
	36–40	47	12.32 ± 8.72			
	≥41	40	9.57 ± 6.92			
Marital status	Unmarried	81	10.47 ± 7.37	1.444	.238	
	Married	121	11.38 ± 8.44			
	Other	8	15.62 ± 15.62			
Rank	Firefighter	66	10.73 ± 7.57	0.145	.865	
	Senior firefighter	80	11.43 ± 9.55			
	Fire sergeant or higher	64	11.38 ± 7.82			
Professional qualification	Level 1 emergency medical technician	33	10.24 ± 7.25	0.823	.482	
	Registered nurse	136	11.79 ± 8.93			
	Level 2 emergency medical technician	29	10.59 ± 7.33			
	Other	12	8.50 ± 7.83			
Length of service (years)	<1	18	8.33 ± 6.71	1.832	.142	
	1–<5	84	10.92 ± 7.58			
	5–<10	56	13.12 ± 10.71			
	≥10	52	10.54 ± 7.12			
Number of dispatches	<5[a]	22	5.27 ± 4.08	22.721	<.001	a < b < c
	5–<10[b]	143	10.51 ± 7.46			
	≥10[c]	45	16.24 ± 10.18			
Monthly salary (10,000 KRW)	<350	67	10.88 ± 9.50	2.169	.117	
	350–<400	65	12.91 ± 8.79			
	≥400	78	10.03 ± 6.85			

B. Post-traumatic stress symptoms

Characteristic	Category	n	M ± SD	t/F	p	Scheffé
Sex	Male	166	16.66 ± 14.49	−0.537	.592	
	Female	44	18.00 ± 15.44			
Age (years)	≤30	49	13.14 ± 12.78	2.131	.097	
	31–35	74	17.14 ± 14.74			
	36–40	47	20.64 ± 14.15			
	≥41	40	16.90 ± 16.53			
Marital status	Unmarried	81	15.32 ± 13.67	1.011	.366	
	Married	121	18.17 ± 15.11			
	Other	8	14.75 ± 17.46			
Rank	Firefighter[a]	66	13.36 ± 12.25	3.898	.023	a < c
	Senior firefighter[b]	80	17.09 ± 13.86			
	Fire sergeant or higher[c]	64	20.45 ± 17.07			
Professional qualification	Level 1 emergency medical technician	33	15.55 ± 15.14	0.653	.582	
	Registered nurse	136	17.95 ± 14.76			
	Level 2 emergency medical technician	29	14.28 ± 13.49			
	Other	12	15.83 ± 15.63			
Length of service (years)	<1	18	11.11 ± 11.19	3.015	.031	
	1–<5	84	14.76 ± 13.30			
	5–<10	56	18.64 ± 14.86			
	≥10	52	20.65 ± 16.63			
Number of dispatches	<5	22	19.77 ± 13.89	2.037	.133	
	5–<10	143	15.55 ± 13.98			
	≥10	45	19.98 ± 16.70			
Monthly salary (10,000 KRW)	<350	67	14.00 ± 14.45	2.057	.130	
	350–<400	65	17.88 ± 14.30			
	≥400	78	18.69 ± 14.95			

C. Burnout

Characteristic	Category	n	M ± SD	t/F	p	Scheffé
Sex	Male	166	45.96 ± 16.35	−3.663	<.001	
	Female	44	56.18 ± 16.89			
Age (years)	≤30	49	43.88 ± 18.59	1.449	.230	
	31–35	74	48.57 ± 15.64			
	36–40	47	50.38 ± 16.53			
	≥41	40	49.73 ± 17.37			
Marital status	Unmarried	81	45.83 ± 16.45	1.266	.284	
	Married	121	49.68 ± 16.56			
	Other	8	47.25 ± 25.97			
Rank	Firefighter[a]	66	44.44 ± 16.61	3.816	.024	a < c
	Senior firefighter[b]	80	47.61 ± 15.78			
	Fire sergeant or higher[c]	64	52.48 ± 17.93			
Professional qualification	Level 1 emergency medical technician	33	48.76 ± 18.45	2.633	.051	
	Registered nurse	136	49.90 ± 16.95			
	Level 2 emergency medical technician	29	42.21 ± 15.11			
	Other	12	40.17 ± 12.46			
Length of service (years)	<1[a]	18	39.06 ± 13.88	6.802	<.001	a, b < d
	1–<5[b]	84	44.33 ± 16.70			
	5–<10[c]	56	50.23 ± 15.97			
	≥10[d]	52	55.02 ± 16.66			
Number of dispatches	<5[a]	22	50.45 ± 12.89	5.064	.007	b < c
	5–<10[b]	143	45.71 ± 16.37			
	≥10[c]	45	54.53 ± 18.86			
Monthly salary (10,000 KRW)	<350[a]	67	44.00 ± 17.35	3.465	.033	a < c
	350–<400[b]	65	48.46 ± 17.08			
	≥400[c]	78	51.32 ± 15.93			

D. Social support

Characteristic	Category	n	M ± SD	t/F	p	Scheffé
Sex	Male	166	48.03 ± 9.51	1.022	.308	
	Female	44	46.34 ± 10.60			
Age (years)	≤30[a]	49	48.88 ± 11.00	3.768	.012	d < b
	31–35[b]	74	49.72 ± 8.93			
	36–40[c]	47	46.47 ± 9.12			
	≥41[d]	40	43.85 ± 9.29			
Marital status	Unmarried	81	48.09 ± 10.34	0.136	.873	
	Married	121	47.46 ± 9.61			
	Other	8	46.75 ± 5.31			
Rank	Firefighter	66	49.89 ± 9.61	2.830	.061	
	Senior firefighter	80	47.21 ± 10.30			
	Fire sergeant or higher	64	45.97 ± 8.85			
Professional qualification	Level 1 emergency medical technician	33	48.12 ± 10.45	0.656	.580	
	Registered nurse	136	47.04 ± 9.65			
	Level 2 emergency medical technician	29	49.24 ± 9.97			
	Other	12	49.83 ± 8.60			
Length of service (years)	<1	18	50.89 ± 11.20	2.516	.059	
	1–<5	84	48.96 ± 9.85			
	5–<10	56	47.16 ± 9.76			
	≥10	52	45.04 ± 8.54			
Number of dispatches	<5	22	49.50 ± 5.18	1.164	.318	
	5–<10	143	47.48 ± 10.29			
	≥10	45	47.42 ± 9.72			
Monthly salary (10,000 KRW)	<350[a]	67	49.40 ± 11.42	4.244	.016	c < a
	350–<400[b]	65	48.89 ± 8.22			
	≥400[c]	78	45.18 ± 8.93			

E. Resilience

Characteristic	Category	n	M ± SD	t/F	p	Scheffé
Sex	Male	166	89.58 ± 11.96	2.598	.010	
	Female	44	84.20 ± 13.07			
Age (years)	≤30	49	90.29 ± 17.47	1.994	.120	
	31–35	74	89.89 ± 9.97			
	36–40	47	85.96 ± 10.65			
	≥41	40	86.47 ± 10.19			
Marital status	Unmarried	81	88.99 ± 14.82	0.515	.598	
	Married	121	87.87 ± 10.66			
	Other	8	91.88 ± 9.23			
Rank	Firefighter	66	91.50 ± 13.78	2.992	.052	
	Senior firefighter	80	87.20 ± 12.61			
	Fire sergeant or higher	64	86.88 ± 9.87			
Professional qualification	Level 1 emergency medical technician	33	87.33 ± 14.43	1.013	.388	
	Registered nurse	136	87.86 ± 11.98			
	Level 2 emergency medical technician	29	90.69 ± 12.64			
	Other	12	92.83 ± 9.45			
Length of service (years)	<1[a]	18	95.89 ± 13.55	4.358	.005	c, d < a
	1–<5[b]	84	90.10 ± 14.02			
	5–<10[c]	56	85.98 ± 10.46			
	≥10[d]	52	85.88 ± 9.48			
Number of dispatches	<5	22	86.82 ± 10.61	0.222	.801	
	5–<10	143	88.71 ± 12.20			
	≥10	45	88.42 ± 13.81			
Monthly salary (10,000 KRW)	<350[a]	67	88.49 ± 14.20	6.942	.001	c < b
	350–<400[b]	65	92.54 ± 12.47			
	≥400[c]	78	85.01 ± 9.31			

Note: Values are presented as mean ± standard deviation. Independent-samples t-tests were used for sex; one-way analysis of variance was used for all other general characteristics, with Scheffé's test for post hoc comparisons. KRW = Korean won. These group-comparison analyses were conducted using the final sample of 210 participants after one influential observation was excluded based on influence diagnostics.

Supplementary Table S2. Sensitivity Analyses of Interaction Effects and Indices of Moderated Mediation in the Initial Sample (N = 211)

A. Interaction effects

Interaction	B	HC3 SE	95% CI	p
WPV × Social support → PTSS	−.020	.016	[−.052, .012]	.211
WPV × Social support → Burnout	−.019	.008	[−.035, −.004]	.016
WPV × Resilience → PTSS	−.012	.011	[−.035, .010]	.273
WPV × Resilience → Burnout	−.023	.011	[−.045, −.001]	.045

B. Indices of moderated mediation

Moderator	Index	Boot SE	Bootstrap 95% CI
Social support	−.015	.011	[−.035, .007]
Resilience	−.009	.008	[−.026, .006]

Note: WPV = workplace violence exposure; PTSS = post-traumatic stress symptoms; HC3 = heteroskedasticity-consistent standard error. The analyses included the same covariates as the primary models. Confidence intervals for the indices of moderated mediation were based on 5,000 bootstrap samples. Social support and resilience were examined in separate PROCESS Model 8 analyses.

Supplementary STROBE Checklist

STROBE Statement—Checklist of Items That Should Be Included in Reports of Cross-Sectional Studies

Manuscript: Workplace Violence Exposure and Burnout among Fire-Service-Based Paramedics in South Korea: The Indirect Association through Post-Traumatic Stress Symptoms and the Moderating Roles of Social Support and Resilience

Section/Topic	Item	STROBE recommendation	Where reported in the manuscript
Title and abstract	1	(a) Indicate the study's design with a commonly used term in the title or the abstract.	Abstract, p. 1
	1	(b) Provide in the abstract an informative and balanced summary of what was done and what was found.	Abstract, p. 1
Introduction—Background/rationale	2	Explain the scientific background and rationale for the investigation being reported.	Introduction, pp. 2–3
Introduction—Objectives	3	State specific objectives, including any prespecified hypotheses.	Section 1.2, p. 4
Methods—Study design	4	Present key elements of study design early in the paper.	Section 2.1, p. 4
Methods—Setting	5	Describe the setting, locations, and relevant dates, including periods of recruitment, exposure, data collection, and data collection context.	Section 2.2, pp. 4–5
Methods—Participants	6	Give the eligibility criteria, and the sources and methods of selection of participants.	Section 2.2, pp. 4–5
Methods—Variables	7	Clearly define all outcomes, exposures, predictors, potential confounders, and effect modifiers. Give diagnostic criteria, if applicable.	Sections 2.3–2.4, pp. 5–7
Methods—Data sources/measurement	8	For each variable of interest, give sources of data and details of methods of assessment (measurement). Describe comparability of assessment methods if there is more than one group.	Section 2.3, pp. 5–7
Methods—Bias	9	Describe any efforts to address potential sources of bias.	Sections 2.2, 2.4, and 4.6, pp. 4–7 and 18–19
Methods—Study size	10	Explain how the study size was arrived at.	Section 2.2, pp. 4–5
Methods—Quantitative variables	11	Explain how quantitative variables were handled in the analyses. If applicable, describe which groupings were chosen and why.	Sections 2.3–2.4, pp. 5–7; Table 1, pp. 8–9
Methods—Statistical methods	12(a)	Describe all statistical methods, including those used to control for confounding.	Section 2.4, p. 7
	12(b)	Describe any methods used to examine subgroups and interactions.	Section 2.4, p. 7
	12(c)	Explain how missing data were addressed.	Section 2.2, p. 5 (mandatory survey items; exclusions described)
	12(d)	If applicable, describe analytical methods taking account of sampling strategy.	Not applicable; convenience sampling was used and no complex sampling design was applied (Section 2.2, pp. 4–5).
	12(e)	Describe any sensitivity analyses.	Section 2.4, pp. 7; Results Sections 3.3.1–3.3.2, pp. 11–13
Results—Participants	13(a)	Report numbers of individuals at each stage of the study—for example, potentially eligible, examined for eligibility, confirmed eligible, included in the study, completing follow-up, and analyzed.	Section 2.2, pp. 4-5
	13(b)	Give reasons for non-participation at each stage.	Section 2.2, p. 5 (two implausible age entries excluded; one influential observation excluded after diagnostics).
	13(c)	Consider use of a flow diagram.	Not used; participant flow is described in Section 2.2, p. 5.
Results—Descriptive data	14(a)	Give characteristics of study participants and information on exposures and potential confounders.	Section 3.1 and Table 1, pp. 8–9; Section 3.2 and Table 2, pp. 9–10
	14(b)	Indicate number of participants with missing data for each variable of interest.	All required survey items were mandatory; Section 2.2, p. 5.
Results—Outcome data	15	Report numbers of outcome events or summary measures.	Section 3.2 and Table 2, pp. 9–10
Results—Main results	16(a)	Give unadjusted estimates and, if applicable, confounder-adjusted estimates and their precision (e.g., 95% confidence interval). Make clear which confounders were adjusted for and why they were included.	Sections 3.2–3.3 and Tables 2–5, pp. 9–15; covariate rationale in Section 2.4, p. 7

Section/Topic	Item	STROBE recommendation	Where reported in the manuscript
	16(b)	Report category boundaries when continuous variables were categorized.	Table 1, pp. 8–9; moderator conditioning described in Section 2.4 and Table 5, pp. 7 and 14–15
	16(c)	If relevant, consider translating estimates of relative risk into absolute risk for a meaningful time period.	Not applicable; no relative-risk estimates were reported.
Results—Other analyses	17	Report other analyses done—for example, analyses of subgroups and interactions, and sensitivity analyses.	Sections 3.3.1–3.3.3 and Tables 3–5, pp. 11–15
Discussion—Key results	18	Summarise key results with reference to study objectives.	Section 4.1, p. 15
Discussion—Limitations	19	Discuss limitations of the study, taking into account sources of potential bias or imprecision. Discuss both direction and magnitude of any potential bias.	Section 4.6, pp. 18–19
Discussion—Interpretation	20	Give a cautious overall interpretation of results considering objectives, limitations, multiplicity of analyses, results from similar studies, and other relevant evidence.	Sections 4.2–4.6, pp. 15–19
Discussion—Generalisability	21	Discuss the generalisability (external validity) of the study results.	Section 4.6, pp. 18–19
Other information—Funding	22	Give the source of funding and the role of the funders for the present study and, if applicable, for the original study on which the present article is based.	Funding statement, p. 20 (Ministry of Education, Glocal University 30 Project at Dong-A University).

Note. This checklist was completed against the revised manuscript. Page references correspond to the current revised manuscript pagination. “Not applicable” is used where a STROBE item does not apply to this analytical cross-sectional study.
